# Supplementary material for: Arabic validation and cross-cultural adaptation of the 5C scale for assessment of COVID-19 vaccines psychological antecedents
Source: PLoS One. 2021 Aug 26;16(8):e0254595. doi: 10.1371/journal.pone.0254595 (PMC8389382; doi:10.1371/journal.pone.0254595)
Supplement: S1 Table — (PDF) [file pone.0254595.s001.pdf]

S1 Table

مقياس 5C لتقييم الدوافع النفسية للتلقحيات (التطعيمات) ضد فيروس كورونا المستجد

الرقم المسلسل للمريض:

تمت هذه الدراسه في عام 2021 بواسطة الدكتورة سمر عبد الحفيظ ، الدكتورة عفت البرازي، الدكتور رامي شعبان ، الدكتورة روني المخزنجي، الدكتور ماجد أسامة، الدكتور عمرو النجار، الدكتور محمد يعقوب، الدكتور حيدر السايح، الدكتورة نشوى الطويل، الدكتورة سولافة القطب، الدكتور رامي محمد غازي تحت اشراف المعهد العالي للصحة العامة – جامعة الإسكندرية . البريد الإلكتروني:
samarabdelhafeez.epid@gmail.com
يخضع استخدام هذه الاستمارة للحصول على موافقة الناشرين

ما هو الغرض من الدراسة؟ يجري تنفيذ هذه الدراسة عن طريق مجموعة من الباحثين العرب. والغرض من ذلك هو ترجمة وقياس مصداقية الاستمارة 5C لتقييم الدوافع النفسية للتلقحيات (التطعيمات) ضد فيروس كورونا المستجد

ماهو المطلوب مني فعله ؟ نحن نريد منك ملأ هذه الاستمارة التلقيحات لفيروس كورونا المستجد وبعض البيانات الخاصة بك .

سرية المعلومات : نحن لا نسأل عن إسمك ولكن سوف يتم الجمع بين إجاباتك مع اجابات المشاركين الاخرين في الإبلاغ عن النتائج التي توصلت إليها الدراسة. وسوف يتم اعتبار أي من المعلومات التي تسمح بالتعرف عليك سريه للغايه بالإضافة إلى أن كل المعلومات المجمعه سوف تستخدم فقط لغرض الدراسه ولن يتم الكشف عنها أو استخدامها لأغراض اخرى قبل الحصول على موافقتك

كيف ستكون المشاركه في الدراسه مفيده لي؟ إن المعلومات التي ستقدمها لنا ستخبرنا عن دوافعك النفسيه وتقبلك للتطعيمات ضد مرض كورونا المستجد

هل لابد لي ان اشارك ؟ أنت لست مضطرا للمشاركه وملأ الاستماره، كما أنه يمكنك رفض الإجابة عن أي سؤال وهذا القرار لن يؤثر على فرصتك في الحصول على الرعاية الصحية.

نشكرك على ملأ هذه الاستمارة

تمت هذه الدراسه في عام 2021 بواسطة الدكتورة سمر عبد الحفيظ ، الدكتورة عفت البرازي، الدكتور رامي شعبان ، الدكتورة روني المخزنجي، الدكتور ماجد أسامة، الدكتور عمرو النجار، الدكتور محمد يعقوب، الدكتور حيدر السايح، الدكتورة نشوى الطويل، الدكتورة سولافة القطب، الدكتور رامي محمد غازي تحت اشراف المعهد العالي للصحة العامة – جامعة الإسكندرية . البريد الإلكتروني:
samarabdelhafeez.epid@gmail.com
يخضع استخدام هذه الاستمارة للحصول على موافقة الناشرين

الجزء الأول: البيانات الشخصية والطبية

A. النوع:

1. ذكر
2. انثي

B. العمر: .... سنة

C. الجنسية:

D. الدولة التي تقيم بها:

E. المؤهل العلمي:

1. ما قبل جامعي
2. مهني / فني
3. جامعي
4. دبلوم / دراسات عليا
5. ماجيستير
6. دكتوراه

F. هل تعاني من أي أمراض مزمنة؟

1. نعم
2. لا

G. هل تعمل بالقطاع الصحي؟

1. نعم
2. لا

H. هل أصبت بالكورونا؟

1. نعم
2. لا
3. لا اعلم

I. هل توفي أحد أقاربك أو معارفك بالكورونا؟

1. نعم
2. لا
3. لا اعلم

تمت هذه الدراسه في عام 2021 بواسطة الدكتورة سمر عبد الحفيظ ، الدكتورة عفت البرازي، الدكتور رامي شعبان ، الدكتورة روني المخزنجي، الدكتور ماجد أسامة، الدكتور عمرو النجار، الدكتور محمد يعقوب، الدكتور حيدر السايح، الدكتورة نشوى الطويل، الدكتورة سولافة القطب، الدكتور رامي محمد غازي تحت اشراف المعهد العالي للصحة العامة – جامعة الإسكندرية . البريد الإلكتروني:
samarabdelhafeez.epid@gmail.com
يخضع استخدام هذه الاستمارة للحصول على موافقة الناشرين

J. هل تعلم بوجود أنواع متعددة من تطعيمات (لقاحات) الكورونا ؟

1. نعم
2. لا

الجزء الثاني: البيانات الخاصة بقياس 5Cلتقييم الدوافع النفسية للتطعيمات ضد مرض كورونا المستجد

من فضلك أجب على هذه الأسئلة الخاصة ب لقاحات أو تطعيمات فيروس كورونا المستجد:

	١ = غير موافق بشدة	٢ = غير موافق بنسبة معتدلة	٣= غير موافق بنسبة قليلة	٤= ليس لي رأي محدد	٥= موافق بنسبة قليلة	٦= موافق بنسبة متوسطة	٧= موافق بشدة
**الثقة**							
**1. أنا واثق تماماً، بأن التطعيمات آمنة.**							
2. التطعيمات (اللقاحات) فعالة.							
3. بالنسبة للتطعيمات (اللقاحات)، أنا واثق بأن السلطات العامة تقرر ما هو أفضل لصالح المجتمع.							
**الرضا**							
**4. التطعيم (اللقاح) غير ضروري لأن الأمراض التي يقي منها لم تعد منتشرة كما في السابق.**							

تمت هذه الدراسه في عام 2021 بواسطة الدكتورة سمر عبد الحفيظ ، الدكتورة عفت البرازي، الدكتور رامي شعبان ، الدكتورة روني المخزنجي، الدكتور ماجد أسامة، الدكتور عمرو النجار، الدكتور محمد يعقوب، الدكتور حيدر السايح، الدكتورة نشوى الطويل، الدكتورة سولافة القطب، الدكتور رامي محمد غازي تحت اشراف المعهد العالي للصحة العامة – جامعة الإسكندرية . البريد الإلكتروني:
samarabdelhafeez.epid@gmail.com
يخضع استخدام هذه الاستمارة للحصول على موافقة الناشرين

5. جهاز مناعتي قوي جداً وأيضًا يحميني من الأمراض.							
6. الأمراض التي يمكن الوقاية منها بالتطعيم (اللقاح) ليست خطيرة بالقدر الكافي لكي يتوجب عليّ الحصول على هذا التطعيم (اللقاح).							
**العوائق**							
7. **يمنعني الضغط اليومي من الحصول على التطعيم (اللقاح).**							
8. بالنسبة لي، من غير الملائم أخذ التطعيم (اللقاح).							
9. أشعر بعدم الارتياح عند زيارة الطبيب، وهذا يجعلني أتجنب أخذ التطعيم (اللقاح).							
**الحسابات**							

**10. عندما أفكر بالحصول على التطعيم (اللقاح)، أقارن ما بين المخاطر والفوائد لاتخاذ أحسن قرار متاح.**							
**11.** بالنسبة لكل تطعيم (لقاح)، أفكر جيدًا في فائدته بالنسبة لي.							
**12.** من المهم جداً بالنسبة لي أن أفهم بشكل كامل كل ما يخص موضوع التطعيمات (اللقاحات) قبل أن أحصل على التطعيم (اللقاح).							
**المسؤولية المشتركة**							
**13. عندما يحصل الجميع على التطعيم (اللقاح)، فأنا لست مضطرًا لأخذ التطعيم (اللقاح) أيضًا. (R)**							
**14.** أقوم بأخذ التطعيم (اللقاح) لأوفّر الحماية لأفراد المجتمع ذوي المناعة الأضعف.							

تمت هذه الدراسه في عام 2021 بواسطة الدكتورة سمر عبد الحفيظ ، الدكتورة عفت البرازي، الدكتور رامي شعبان ، الدكتورة روني المخزنجي، الدكتور ماجد أسامة، الدكتور عمرو النجار، الدكتور محمد يعقوب، الدكتور حيدر السايح، الدكتورة نشوى الطويل، الدكتورة سولافة القطب، الدكتور رامي محمد غازي تحت اشراف المعهد العالي للصحة العامة – جامعة الإسكندرية . البريد الإلكتروني:
samarabdelhafeez.epid@gmail.com
يخضع استخدام هذه الاستمارة للحصول على موافقة الناشرين

**15.** التطعيم (اللقاح) عمل جماعي لمنع انتشار الأمراض.							

من فضلك قيم الى أي مدى أنت غير موافق/ موافق على الجمل المذكورة (١= غير موافق بشدة، ٢= غير موافق بنسبة متوسطة، ٣= غير موافق بنسبة قليلة، ٤= ليس لي رأي محدد، ٥= موافق بنسبة قليلة بشدة، ٦= موافق بنسبة متوسطة، ٧= موافق بشدة).

التصنيف: متوسط درجات كل مقياس فرعي. العنصر ذو (R) تكويدعكسي. للمقياس القصير ، استخدم العناصر الغامقة.

تمت هذه الدراسه في عام 2021 بواسطة الدكتورة سمر عبد الحفيظ ، الدكتورة عفت البرازي، الدكتور رامي شعبان ، الدكتورة روني المخزنجي، الدكتور ماجد أسامة، الدكتور عمرو النجار، الدكتور محمد يعقوب، الدكتور حيدر السايح، الدكتورة نشوى الطويل، الدكتورة سولافة القطب، الدكتور رامي محمد غازي تحت اشراف المعهد العالي للصحة العامة – جامعة الإسكندرية . البريد الإلكتروني:
samarabdelhafeez.epid@gmail.com

يخضع استخدام هذه الاستمارة للحصول على موافقة الناشرين
