# Supplementary material for: Arabic validation and cross-cultural adaptation of the 5C scale for assessment of COVID-19 vaccines psychological antecedents
Source: PLoS One. 2021 Aug 26;16(8):e0254595. doi: 10.1371/journal.pone.0254595 (PMC8389382; doi:10.1371/journal.pone.0254595)
Supplement: S2 Table — (PDF) [file pone.0254595.s002.pdf]

S2 Table. Translation table.

translated version	English version
Confidence	
	**I am completely confident that vaccines are safe.**
	Vaccinations are effective.
	Regarding vaccines, I am confident that public authorities decide in the best interest of the community.
Complacency	
	**Vaccination is unnecessary because vaccine-preventable diseases are not common anymore.**
	My immune system is so strong, it also protects me against diseases.
	Vaccine-preventable diseases are not so severe that I should get vaccinated.
Constraints	
	**Everyday stress prevents me from getting vaccinated.**
	For me, it is inconvenient to receive vaccinations.
	Visiting the doctor's makes me feel uncomfortable; this keeps me from getting vaccinated.
Calculation	
	**When I think about getting vaccinated, I weigh benefits and risks to make the best decision possible.**
	For each and every vaccination, I closely consider whether it is useful for me.
	It is important for me to fully understand the topic of vaccination, before I get vaccinated.
Collective responsibility	
	**When everyone is vaccinated, I don't have to get vaccinated, too. (R)**
	I get vaccinated because I can also protect people with a weaker immune system.
	Vaccination is a collective action to prevent the spread of diseases.

Instruction: "Please evaluate how much you disagree or agree with the following statements." (1 = strongly disagree, 2 = moderately disagree, 3 = slightly disagree, 4 = neutral, 5 = slightly agree, 6 = moderately agree, 7 = strongly agree). Scoring: mean scores of each sub-scale. Item with (R) is reverse-coded. For the short scale use bold items.
