# Supplementary material for: Arabic validation and cross-cultural adaptation of the 5C scale for assessment of COVID-19 vaccines psychological antecedents
Source: PLoS One. 2021 Aug 26;16(8):e0254595. doi: 10.1371/journal.pone.0254595 (PMC8389382; doi:10.1371/journal.pone.0254595)
Supplement: S3 Table — (PDF) [file pone.0254595.s003.pdf]

S3 Table: Inter-item correlations of the Arabic version of the 5C scale

**Confidence**			**Complacency**			**Constraints**			**Calculation**			**Collective responsibility**		
	Q2	Q3		Q5	Q6		Q8	Q9		Q11	Q12		Q14	Q15
Q1	0.81(*P*< 0.001)	0.58(*P*< 0.001)	Q4	0.50(*P*< 0.001)	0.47(*P*< 0.001)	Q7	0.35(*P*< 0.001)	0.28(*P*< 0.001)	Q10	0.58(*P*< 0.001)	0.46(*P*< 0.001)	Q13	0.56(*P*< 0.001)	0.58(*P*< 0.001)
Q2		0.51(*P*< 0.001)	Q5		0.40(*P*< 0.001)	Q8		0.53(*P*< 0.001)	Q11		0.59(*P*< 0.001)	Q14		0.74(*P*< 0.001)
